# Levels of Breast Milk MicroRNAs and Other Non-Coding RNAs Are Impacted by Milk Maturity and Maternal Diet

**DOI:** 10.3389/fimmu.2021.785217

**Published:** 2022-01-14

**Authors:** Steven D. Hicks, Alexandra Confair, Kaitlyn Warren, Desirae Chandran

**Affiliations:** Division of Academic General Pediatrics, Department of Pediatrics, Penn State College of Medicine, Hershey, PA, United States

**Keywords:** breast milk, RNA, nutrition, miRNA – microRNA, non-coding RNAs

## Abstract

There is emerging evidence that non-coding RNAs (ncRNAs) within maternal breast milk (MBM) impart unique metabolic and immunologic effects on developing infants. Most studies examining ncRNAs in MBM have focused on microRNAs. It remains unclear whether microRNA levels are related to other ncRNAs, or whether they are impacted by maternal characteristics. This longitudinal cohort study examined 503 MBM samples from 192 mothers to: 1) identify the most abundant ncRNAs in MBM; 2) examine the impact of milk maturity on ncRNAs; and 3) determine whether maternal characteristics affect ncRNAs. MBM was collected at 0, 1, and 4 months post-delivery. High throughput sequencing quantified ncRNAs within the lipid fraction. There were 3069 ncRNAs and 238 microRNAs with consistent MBM presence (≥10 reads in ≥10% samples). Levels of 17 ncRNAs and 11 microRNAs accounted for 80% of the total RNA content. Most abundant microRNAs displayed relationships ([R]>0.2, adj p< 0.05) with abundant ncRNAs. A large proportion of ncRNAs (1269/3069; 41%) and microRNAs (206/238; 86%) were affected by MBM maturity. The majority of microRNAs (111/206; 54%) increased from 0-4 months. Few ncRNAs and microRNAs were affected (adj p < 0.05) by maternal age, race, parity, body mass index, gestational diabetes, or collection time. However, nearly half of abundant microRNAs (4/11) were impacted by diet. To our knowledge this is the largest study of MBM ncRNAs, and the first to demonstrate a relationship between MBM microRNAs and maternal diet. Such knowledge could guide nutritional interventions aimed at optimizing metabolic and immunologic microRNA profiles within MBM.

## 1 Introduction

Maternal breast milk (MBM) is the optimal nutrition source for developing infants (1). MBM contributes to immunologic function in the newborn period (2, 3). This occurs through passive transfer of maternal factors such as secretory IgA, lactoferrin, and lysozymes, which provide short-term protection to the infant gut and upper respiratory tract (4, 5). These factors may contribute to lower rates of gastroenteritis, nasopharyngitis, and otitis observed in breastfed infants relative to formula-fed peers (6–8). Importantly, MBM also confers long-term immunologic benefits. For example, several studies have found associations of MBM exposure in infancy with reductions in autoimmune conditions, enhanced vaccine responsiveness, and atopy protection in later childhood (9–11). The precise factors within MBM that promote these sustained benefits are poorly understood. Many studies have focused on the relationship between traditional immunologic components (i.e., immunoglobins, cytokines, lymphocytes) and the benefits of breast feeding (12, 13). However, there is emerging evidence that MBM is an abundant source of non-coding ribonucleic acids (ncRNAs) (14–20). The physiologic role of ncRNAs in immune function suggests that their presence in MBM may contribute to infant immune development and sustained immunologic health (21–24).

A recent explosion in ncRNA studies have demonstrated that these nucleic acids play a critical role in translational regulation and contribute to human complexity (25–27). Families of ncRNAs include ribosomal RNA (rRNA) and transfer RNA (tRNA), which facilitate the translation of messenger RNA (mRNA) into proteins (28). Increasing interest in ncRNAs has shed light on additional regulatory elements, such as microRNA (miRNA), small nucleolar RNA (snoRNA), and long ncRNA (lncRNA), which collectively regulate steps between transcription and translation through epigenetic modification of DNA, or direct interaction with mRNA (29–31). Of all ncRNAs, miRNAs have been most studied in MBM (15–24). miRNAs are often packaged within protective vesicles (32), making them stable in MBM and capable of transfer to the infant gut, where they may be absorbed and functionally incorporated by cells lining the gut (33, 34). Animal studies suggest nutritional miRNA influences the immune system through immune-modulation (35, 36). Thus, MBM miRNA exposure may mitigate infant risk for atopy and infection.

A recent review examining ncRNA expression across 30 human MBM studies comprehensively summarized the state of knowledge in this nascent field (37). MBM contains high quantities of miRNA (38). The majority of miRNAs in MBM originate from mammary epithelium (15, 39). miRNA profiles are generally consistent across skim, lipid, and cellular fractions (14, 15), and high-throughput sequencing approaches have shown that a wide variety of ncRNAs exist in MBM. These studies suggest a small subset of miRNAs predominate (miR-148a-3p, miR-30a/d-5p, miR-22-3p, miR-146b-5p, miR-200a/c-3p, and let-7a-5p) (15–18). However, miRNA composition may vary with sample handling, RNA extraction, and quantification techniques (40–42). Many studies have reported that miRNA profiles are impacted by maternal factors such as age, parity, BMI, stress, and smoking (37). However, a limitation of previous investigations has been small sample sizes, which prevent firm conclusions about relationships between MBM miRNAs and maternal characteristics.

The goal of this longitudinal cohort study (NCT04017520) was to characterize the relationship between MBM RNA levels and maternal medical/demographic factors. We hypothesized that MBM miRNA levels would fluctuate with milk maturity and be influenced by maternal diet. To our knowledge, this is the largest study of MBM RNA levels (503 samples). It provides a novel opportunity to assess MBM ncRNA relationships with numerous maternal characteristics, and characterize MBM levels of ncRNA features that have received less research attention relative to miRNAs.

## 2 Methods

This study was approved by the Independent Review Board at the Penn State College of Medicine (STUDY00008657). Written informed consent was obtained from all participant at enrollment.

### 2.1 Participants

This study involved a longitudinal cohort of 221 mother/infant dyads. Targeted recruitment occurred following delivery at the Penn State Hershey Medical Center (PSHMC), and at initial well child visits to the PSHMC outpatient pediatric clinics. Inclusion criteria were: mothers (ages 19-42 years) who delivered at term (>35 weeks), and intended to breastfeed beyond four months. Exclusion criteria were: 1) maternal morbidities that could affect ability to breastfeed or influence MBM RNA composition (e.g. cancer, drug addiction, human immunodeficiency virus infection); 2) plan for infant adoption, or family move outside Central Pennsylvania within 12 months; 3) presence of neonatal condition that could significantly affect ability to breastfeed (e.g. cleft lip, metabolic disease, or NICU admission >7 days); and 4) families seeking pediatric care outside PSHMC. Between April, 2018 and October, 2020 research staff screened 2487 potential participants through review of the electronic medical record, approached 359 eligible participants (14.4%), and enrolled 221 participants (61.5% of those eligible) (**Figure S1, Supplementary Material**). All participants had access to on-site lactation support for the duration of the study.

### 2.2 Data and Sample Collection

#### 2.2.1 Medical and Demographic Data

Medical/demographic data were collected from participating mothers across three time points: 0 months (4 ± 2 days) post-delivery, 1 month (39 ± 11 days) post-delivery, and 4 months (128 ± 8 days) post-delivery. These time-points were chosen for ease of collection (alignment with well child visits) and to specifically capture important changes in miRNA content that might occur across the period when health benefits attributed to breastfeeding occur (43). The following medical and demographic information was recorded through survey (and confirmed by chart review) at the time of enrollment: maternal age, maternal race, parity, maternal pre-pregnancy body mass-index (BMI; kg/m^2^), presence/absence of gestational diabetes, history of maternal tobacco use, and previous breastfeeding duration. At the time of each MBM sample collection, the following information was collected: time of day (morning, 7:00 AM – 11:59 AM; afternoon, 12:00 PM – 5:59 PM; night, 6:00 PM – 6:59 AM), and maternal diet. Maternal diet was assessed using the Dietary Screener Questionnaire (44). Standardized scoring of this tool, as established by the National Health and Nutrition Examination Survey, was used to determine average maternal intake of calcium, dairy, fruits, sugar, and vegetables for the 30 day period prior to MBM collection. Missing survey data (24/9030, 0.2%) was imputed using the group median value.

#### 2.2.2 Milk Samples

**Figure 1:**
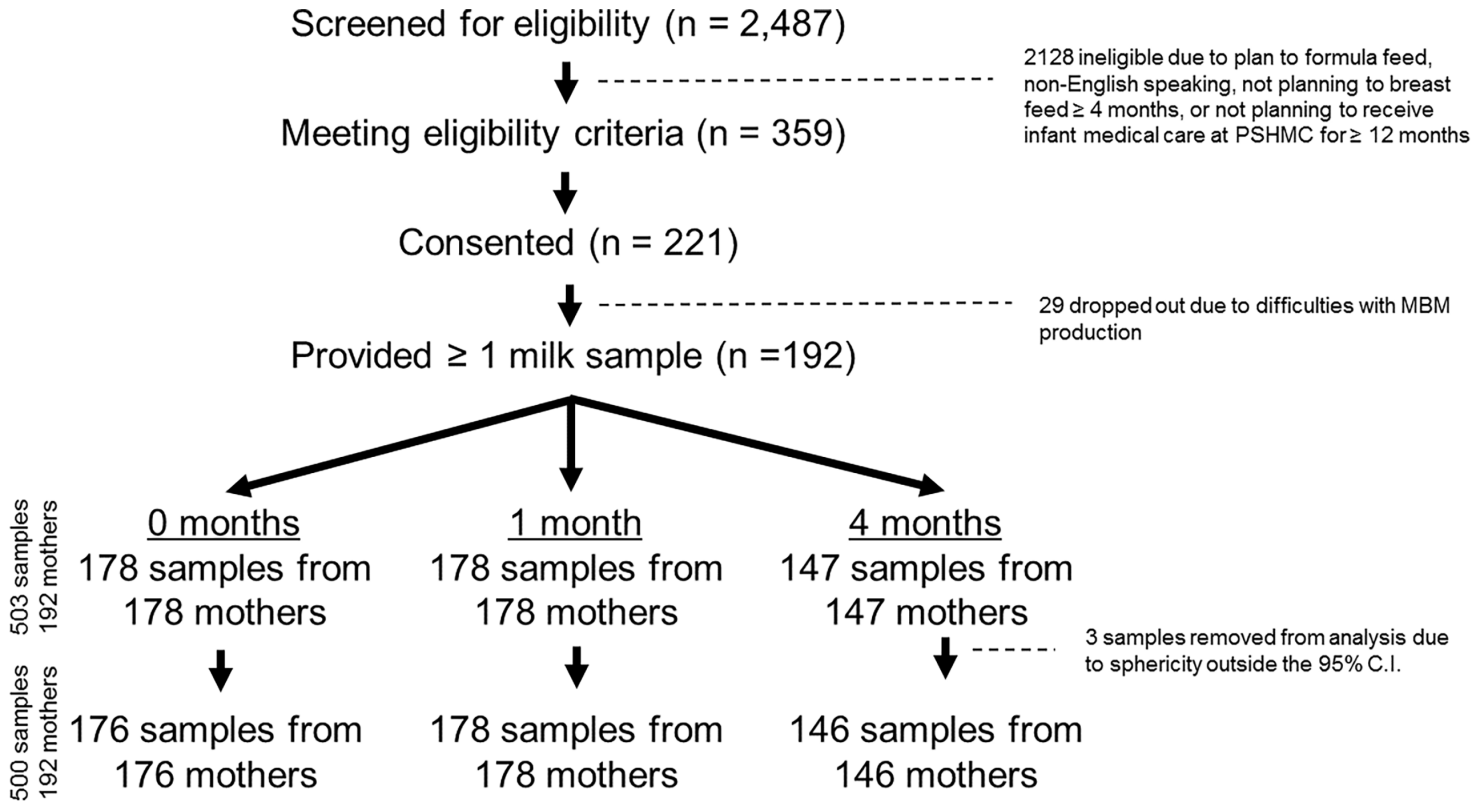
CONSORT Diagram. There were 2,487 individuals screened for eligibility, and 359 eligible mothers were approached. 221 mothers consented to participate, and 192 of these mothers ultimately provided at least 1 milk sample. In total, 503 milk samples were collected (178 samples at 0 months, 178 samples at 1 month, and 147 samples at 4 months post-delivery). Three samples were removed after sphericity analysis, leaving 500 total samples for the final analysis.

MBM samples were collected at 0, 1, and 4 months post-delivery. Twenty-nine mothers dropped out of the study prior to MBM collection due to difficulties with MBM production (29/221, 13%) (**Figure 1**). In addition, 14 mothers declined to provide MBM at 0 months due to concerns about milk supply (14/192, 7%). Another 14 mothers ceased breastfeeding prior to 1 month (14/192, 7%). There were 45 mothers who ceased breastfeeding prior to 4 months (45/192, 23%). In total, this yielded 503 milk samples from 192 unique mothers for RNA sequencing. Seventeen women provided 1 sample, 39 women provided 2 samples, and 136 women provided 3 samples. MBM (1-5 ml) was manually expressed from a sterilized nipple surface (with soap and water) into RNAse-free tubes prior to feeding (i.e., fore-milk). Our previous investigations indicate minimal differences in miRNA content exist between fore- and hind-milk (45). However, we exclusively utilized pre-feed samples to minimize confounding (15). To control for differences between breasts, mothers were instructed to utilize the same breast for MBM sampling at each time-point. Samples were immediately transferred to -20°C, underwent 1 freeze-thaw cycle for aliquoting, and were placed at -80°C while awaiting RNA extraction. Prior studies have found minimal impacts of freeze-thaw cycles on milk miRNAs (46).

### 2.3 Sample Processing

To eliminate batch effects from RNA extraction, repeat samples from mother-infant dyads were processed simultaneously whenever possible. Samples were spun for 20 min at 4°C at 800 rpm to separate lipid, skim, and cellular fractions. For each sample, 50 μl of the lipid fraction was used for RNA extraction. We focused on the lipid fraction of MBM based upon findings from our lab and others, demonstrating that this fraction contains robust concentrations of immunologic miRNAs with high potential for maternal-infant transfer (16, 17, 20, 46). RNA purification was performed using a Norgen Circulating and Exosomal RNA Purification Kit (Norgen Biotech; Ontario, Canada), as previously described (17). RNA quality was confirmed on an Agilent Bioanalyzer 2100 (Agilent, Santa Clara, CA, USA). RNA was sequenced at the SUNY Molecular Analysis Core using the Illumina TruSeq Small RNA Prep protocol and a NextSeq500 instrument (Illumina; San Diego, CA, United States) at a targeted depth of ten million, 50 base, single-end reads per sample. Reads were aligned to the hg38 build of the human genome using Partek Flow (Partek; St. Louis, MO, United States) and the Bowtie2 aligner. RNAseq was selected to permit interrogation of all known ncRNAs within the human genome, and allow comparison of miRNA features with other ncRNAs. Mature miRNA counts within each sample were quantified with miRBase v22, while other ncRNA features (e.g., rRNAs, tRNAs, snoRNAs) were quantified with Ensemble Transcripts v95. Across all 503 samples, there were 3.21x10^9^ total reads, and 1.86x109 reads were aligned to the human genome (mean: 3.7x10^8^ reads/sample). Read quality was consistent across samples (mean quality score: 34.65, range: 34.20 – 35.06; **Table S1, Supplementary Material**). Individual samples were examined for sphericity on principal components analysis. Three samples that exceeded the 95% confidence interval were excluded (**Figure 1**). The RNA features with consistent detection (raw read counts ≥ 10 in ≥ 10% of samples) were median normalized and mean-center scaled.

### 2.4 Statistical Analysis

Medical and demographic characteristics of mothers contributing samples at 0, 1, and 4 months were compared using Student’s t-test (continuous variables) or chi-square test (nominal variables). A repeated measures, within-subject non-parametric analysis of variance (ANOVA) with Benjamini-Hochberg False Discovery Rate (FDR) correction was used to compare RNA levels across time-points (0, 1, and 4 months) from the mothers who provided a sample at every time-point. Because large numbers of RNAs were impacted by milk maturity (i.e., 0, 1, or 4 month collection), the relationships between maternal characteristics and RNA levels were assessed separately for each time point using a non-parametric Mann-Whitney U-test (dichotomous variables), a non-parametric Kruskal-Wallis ANOVA (nominal/ordinal variables), or a Spearman Rank test (continuous variables). FDR correction was applied to all results. Next, for the miRNAs that were most abundant in MBM (i.e., the 11 miRNAs representing 80% of all miRNA reads), mixed effects models were fit by restricted maximum likelihood to examine the impact of maternal characteristics and milk maturity. Eleven models were created, with a single miRNA as the dependent variable, participant ID as the clustering variable, and maternal characteristics as covariates. Model fit was measured by Aikake and Bayesian Information Criterion, and conditional R^2^. Model significance was reported using the likelihood ratio test. Effects of maternal characteristics and milk collection time were assessed with fixed effects omnibus tests. Putative functions of the most abundant miRNAs were explored in DIANA miRPath v3 (47), through identification of likely mRNA targets (Targetscan score > 0.85). Enrichment of Kyoto Encyclopedia Genes and Genomes (KEGG) pathways by these targets was compared to that expected by chance using a Fisher’s exact test with FDR correction. Relationships between the most abundant miRNAs and the most abundant ncRNAs were interrogated with Spearman Rank correlation. All analyses were performed in Metaboanalyst v5.0 or Jamovi v2.0 (48, 49).

## 3 Results

### 3.1 Participant Characteristics

Participating mothers were mostly white (152/192, 79%), had an average age of 30 years (range: 19 – 41 years), and tended to have given birth once previously (median parity: 2, range: 0-9) (**Table 1**). Few had previously used tobacco (28/192, 15%) or experienced gestational diabetes (21/192, 11%). The average BMI of participating mothers was 27.6 kg/m^2^ (± 6.6). Nearly half (99/192, 52%) had previously breastfed over four months, whereas 78 (41%) had never previously breastfed. Most milk samples were collected during the morning (239/500 samples, 48%) or the afternoon (216/500 samples, 43%). On average, 0 month samples were collected on day 4 (± 2), 1 month samples were collected on day 39 (± 11), and 4 month samples were collected on day 128 (± 8). There was no difference in medical/demographic characteristics between mothers contributing samples at 0, 1, or 4 months.

**Table 1:** Participant and sample characteristics.

	All (n=192)	0 months (n=176)	1 month (n=178)	4 months (n=146)
Age, years, mean (range)	30 (19-41)	30 (19-41)	30 (19-41)	31 (20-41)
*Race, n (%)*				
White	152 (79)	139 (79)	143 (80)	121 (83)
Black	12 (6)	11 (6)	9 (5)	5 (3)
Asian	11 (6)	10 (6)	10 (6)	9 (6)
Bi-racial	7 (4)	6 (3)	6 (3)	5 (3)
Other	10 (5)	10 (6)	10 (6)	6 (4)
Parity, n, median (range)	2 (0-9)	2 (0-9)	2 (0-9)	2 (0-7)
BMI, kg/m2 (mean, SD)	27.6 (6.6)	27.6 (6.7)	27.1 (6.1)	26.8 (6.2)
Gestational diabetes, n (%)	21 (11)	19 (11)	20 (11)	16 (11)
Previous tobacco use, n (%)	28 (15)	26 (15)	22 (12)	17 (12)
*Previous breastfeeding duration, n (%)*				
Never breastfed	78 (41)	69	71	52
0-1 months	4 (2)	4	2	2
1-2 months	4 (2)	4	3	0
2-4 months	7 (3)	6	5	5
> 4 months	99 (52)	93	97	87
Milk maturity, days post-delivery, mean (SD)	53 (0-163)	4 (2)	39 (11)	128 (8)
*Collection time, n (%)*				
Morning (7:00am-11:59am)	239 (48)	87 (50)	82 (46)	70 (48)
Afternoon (12:00pm-5:59pm)	216 (43)	62 (35)	91 (51)	63 (43)
Night (6:00pm-6:59am)	45 (9)	27 (15)	5 (3)	13 (9)
*Diet, mean DSQ score (SD)*				
Dairy	1.76 (0.6)	1.89 (0.6)	1.80 (0.6)	1.64 (0.5)
Calcium	1006 (190)	1054 (196)	1018 (201)	969 (177)
Fruit	1.08 (0.4)	1.15 (0.5)	1.08 (0.4)	1.04 (0.4)
Sugar	17.7 (4.9)	18.6 (7)	17.6 (5)	16.6 (4)
Vegetable	1.52 (0.3)	1.56 (0.3)	1.52 (0.3)	1.58 (0.3)

BMI, Body mass index; DSQ, Dietary survey questionnaire.

### 3.2 Milk RNA Profiles

**Figure 2:**
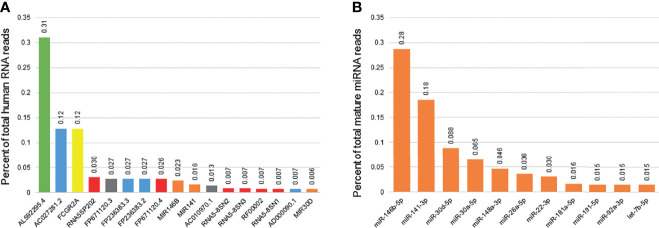
Most consistently expressed small RNAs in human breast milk. The bar graph displays the 17 ncRNAs most consistently detected in human breast milk samples from 192 women **(A)**. These 17 ncRNAs account for 80% of the total ncRNA reads detected. They include one mitochondrial RNA (green), four long non-coding RNAs (blue), one protein coding genes (yellow), five ribosomal RNAs (red), two miscellaneous pseduogenes (grey), and three microRNAs (miRNAs; orange). Percentage of total reads is displayed for each ncRNA. The second bar graph displays the 11 miRNAs most consistently detected in human breast milk **(B)**. These 11 miRNAs account for 80% of the total mature miRNA reads detected.

Among the 57,152 RNAs interrogated using Ensemble v95, there were 52,309 unique RNAs present in at least one sample. There were 3069 RNAs with consistent presence (raw read counts ≥ 10 in ≥ 10% of samples) (**Table S2, Supplementary Material**). Common sub-classes of ncRNAs with consistent presence included miRNAs (203 miRNAs, 1.19x10^8^ reads), rRNAs (161 rRNAs, 1.45x10^8^ reads) and snoRNAs (95 snoRNAs, 6.03x10^6^ reads). Of the 3069 RNAs consistently detected, 17 ncRNAs accounted for 80% of the total RNA reads (**Figure 2**). Levels of succinate dehydrogenase cytochrome b560 subunit mitochondrial isoform 4 (AL592295.4) were the most abundant (4.07x10^8^ raw reads, 31.0% of all reads), while levels of mitochondrially encoded tRNA glutamine (MT-TQ) were the most consistent (present in 499/500 samples, 99.8%).

The 2,652 mature miRNAs interrogated by miRBase v22 accounted for 2.15x10^8^ total reads. There were 1755 unique miRNAs present in at least one sample. There were 238 miRNAs with consistent presence (raw read counts ≥ 10 in ≥ 10% of samples) (**Table S3, Supplementary Material**). Of those consistently detected, 11 miRNAs accounted for 80% of the total miRNA reads (**Figure 1**). Levels of miR-146b-5p were the most abundant (6.18x10^7^ raw reads, 28.7% of all reads), while levels of miR-148a-3p were the most consistent (present in 500/500 samples, 100%).

### 3.3 Relationships of MBM RNA Levels With Maternal Characteristics

#### 3.3.1 Milk Maturity

**Figure 3:**
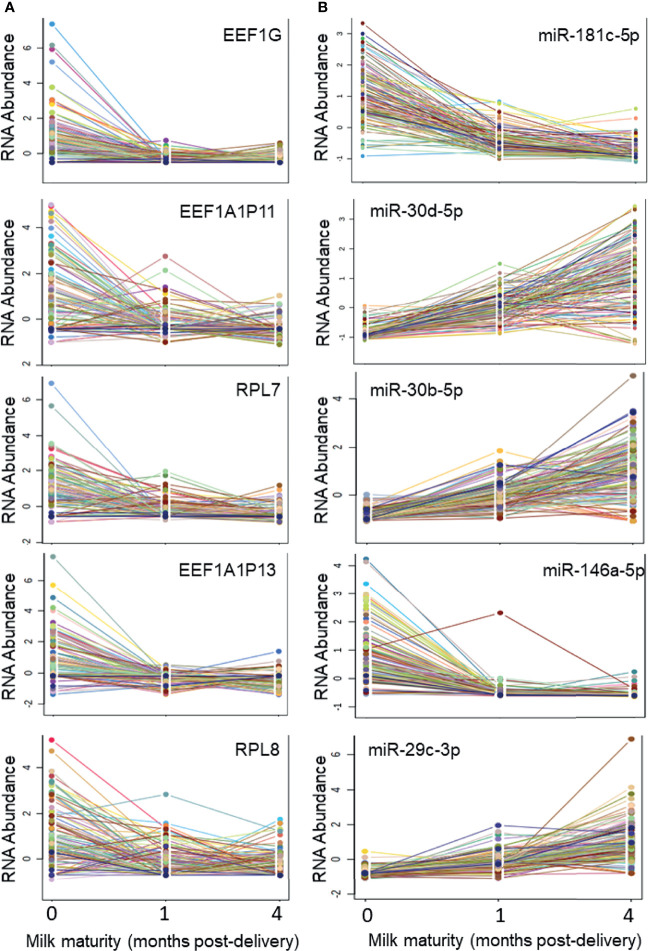
Five ncRNAs and five miRNAs most highly impacted by milk maturity The string plots display changes in five non-coding RNAs (ncRNAs; **A**) and five microRNAs (miRNAs; **B**) most significantly (adj p < 0.05) impacted by milk maturity (i.e., collection at 0, 1, or 4 months post-delivery). The majority of miRNAs increased with increasing milk maturity, whereas a greater proportion of other ncRNA classes decreased with increasing milk maturity.

There were 1269 ncRNAs that displayed an effect (adj. p<0.05) of milk maturity (i.e., collection at 0, 1, or 4 months post-delivery) (**Table S4, Supplementary Material**). The majority (698/1269; 55%) decreased from 0 to 4 months. The five ncRNAs most significantly impacted by milk maturity included three components of the eukaryotic elongation factor-1 complex (EEF1, involved in tRNA delivery), and two rRNAs (**Figure 3**). Of the 17 most abundant ncRNAs, 10 were affected by milk maturity (FP671120.3, FP236383.3, FP236383.2, FP671120.4, AC010970.1, RNA5-8SN2, RNA5-8SN3, RF00002, RNA5-8SN1, MIR30D). There were 206 mature miRNAs that displayed an effect of milk maturity (**Table S4, Supplementary Material**). The majority (111/206, 54%) increased from 0 to 4 months. The five miRNAs most significantly impacted by milk maturity included miR-181c-5p, miR-30d-5p, miR-20b-5p, miR-146a-5p, and miR-29c-3p (**Figure 3**). Of the 11 miRNAs that accounted for 80% of all miRNA content, 10 (90%) were affected by milk maturity.

#### 3.3.2 Maternal Diet

Levels of 3 ncRNAs were associated with dairy intake (DIS3, R=0.31, adj. p=0.034; DMXL1, R=0.30, adj p=0.034; TRPM2, R=0.030, adj p=0.036) at 0 months post-delivery (**Figure S2, Supplementary Material**). No ncRNAs were associated with dairy intake at 1 or 4 months (**Table S5, Supplementary Material**). There was 1 ncRNA associated with fruit intake (CRIP2, R=0.32, adj p=0.023) at 0 months, and 1 ncRNA associated with fruit intake at 1 month (HNRNPA1, R=0.33, adj p=0.0057). None were associated with fruit intake at 4 months. Levels of 1 ncRNA were associated with sugar intake (EIF3F, R=0.38; adj p=0.00022) at 0 months, but none were associated with sugar intake at 1 or 4 months. There were no ncRNAs associated with calcium or vegetable intake at 0, 1, or 4 months. Levels of mature miRNAs displayed few associations with dietary factors (**Table S6, Supplementary Material**). Levels of miR-374b-3p were directly associated (R=0.27, adj p=0.020) with calcium intake at 1 month (**Figure S2, Supplementary Material**).

#### 3.3.3 Maternal Breastfeeding Experience

No ncRNAs displayed an effect of prior breastfeeding duration (**Table S7, Supplementary Material**). Notably, however, at 0 months many more ncRNAs (366 ncRNAs) displayed nominal differences (raw p<0.05) between mothers with/without prior breastfeeding experience than at 4 months (38 ncRNAs). There was an effect of previous breast feeding duration on levels of 1 miRNA at 0 months, 6 miRNAs at 1 month, and 1 miRNA at 4 months post-delivery. Levels of miR-885-5p, miR-196a-5p, and miR-516a-5p were highest among mothers who had previously breastfed > 4 months (**Figure S3, Supplementary Material**).

#### 3.3.4 Maternal Race, Tobacco Use, and Parity

Few ncRNAs or miRNAs displayed an effect of maternal race (**Table S8, Supplementary Material**), tobacco use history (**Table S9, Supplementary Material**), or parity (**Table S10, Supplementary Material**). At 0 months, 2 ncRNAs (RNVU1-18, *x*2 = 27.9, adj p=0.035; RNU1-27P, *x*2 = 26.7, adj p=0.035) displayed an effect of maternal race (**Table S8, Supplementary Material**). At 0 months, levels of miR-196a-5p were lower (*x*2 = 33.4, adj p=7.2E-4) in the MBM of mothers who were delivering their first baby (**Figure S4, Supplementary Material**). At 0 months, levels of miR-196a-5p were also lower among former smokers (adj p=0.013, Log2 fold change = 0.118).

#### 3.3.5 Maternal BMI and Gestational Diabetes

Levels of ncRNAs were not associated with maternal BMI at 0, 1, or 4 months post-delivery (**Table S11, Supplementary Material**). Levels of miR-766-3p were associated with maternal BMI at 1 month (R=0.26, adj p=0.042). No other miRNAs were associated with maternal BMI at 0, 1, or 4 months. Neither ncRNAs nor mature miRNAs displayed an effect of gestational diabetes at 0, 1, or 4 months (**Table S12, Supplementary Material**).

#### 3.3.6 Maternal Age and Time of Day

Levels of ncRNAs were not associated with maternal age at 0, 1, or 4 months post-delivery (**Table S13, Supplementary Material**). Levels of miR-196a-5p were associated with maternal age (R = 0.28, adj p = 0.010) at 1 month (**Figure S4, Supplementary Material**). No other miRNAs were associated with maternal age at 0, 1, or 4 months. Neither ncRNAs nor mature miRNAs displayed an effect of time of day at 0, 1, or 4 months (**Table S14, Supplementary Material**).

### 3.4 Analysis of Abundant MBM miRNAs

#### 3.4.1 Mixed Effects Models

Maternal factors impacting the 11 miRNAs most abundant in MBM were examined using a mixed effects model (**Table S15, Supplementary Material**). Total variance explained through both fixed and random effects was highest for miR-30d-5p (conditional R^2^ = 0.619) and miR-22-3p (conditional R^2^ = 0.409). Addition of maternal characteristics significantly improved the model (LRT p < 0.05) for four miRNAs (miR-146b-5p, miR-26a-5p, miR-181a-5p, let-7b-5p). Omnibus tests of fixed effects identified 10 miRNAs with an effect of milk maturity, 3 miRNAs with an effect of dairy intake, 1 miRNA with an effect of calcium intake, and 1 miRNA with an effect of gestational diabetes.

#### 3.4.2 Functional Analysis

The 11 miRNAs most abundant in MBM displayed target enrichment for 1512 messenger RNAs. Collectively, these targets represented 18 physiologic KEGG pathways with greater frequency than expected by chance alone (**Table S16, Supplementary Material**). Physiologically relevant pathways targeted by MBM miRNAs were: estrogen signaling (15 genes, 6 miRNAs, adj p=0.045), regulation of actin cytoskeleton (27 genes, 7 miRNAs, adj p=0.031), cGMP-PKG signaling (27 genes, 7 miRNAs, 0.0008), and transforming growth factor-beta signaling (12 genes, 6 miRNAs, adj p=0.0014).

#### 3.4.3 Relationships of miRNAs and ncRNAs

**Figure 4:**
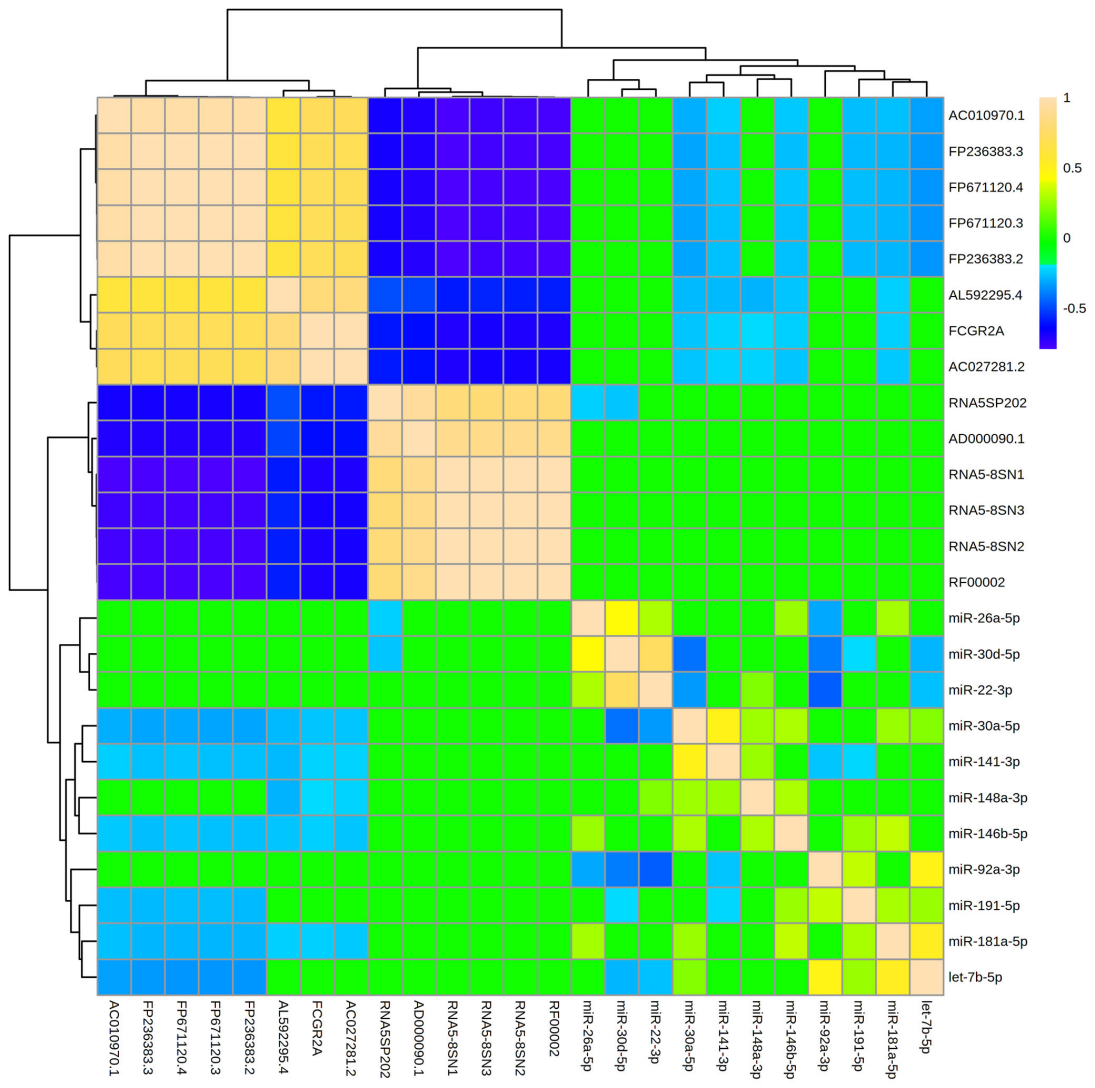
Levels of abundant ncRNAs and miRNAs in maternal breast milk are strongly related. The hierarchical clustering heatmap displays relationships (measured by Spearman rank testing) between the most abundant microRNAs (miRNAs) and non-coding RNAs (ncRNAs) within maternal breast milk. Strong indirect relationships are shown in dark blue. Strong direct relationships are shown in tan. A ward clustering algorithm clustered miRNAs and ncRNAs by relatedness. The 11 miRNAs displayed 30 significant relationships ([R]>0.20, adj p < 0.05) with one another, and 47 relationships with the abundant ncRNAs. The miRNA-miRNA relationships were generally stronger in magnitude than miRNA-ncRNA relationships. Note that miRNAs were generally inversely associated with other ncRNA classes (i.e., blue relationships).

The 11 miRNAs that were most abundant in MBM displayed 30 significant relationships ([R] > 0.2, adj p<0.05) with one another (**Figure 4**). There were 20 direct, and 10 indirect relationships. The strongest relationships were observed between miR-22-3p and miR-30d-5p (R = 0.72, adj p = 1.2x10^-14^), and between miR-92a-3p and miR-22-3p (R = -0.45, adj p = 3.2x10^-6^). The miRNAs displayed similar numbers of significant relationships with other ncRNAs (47). However, all 47 relationships were indirect, and the strength of these relationships was generally weaker than miRNA-miRNA relationships. The strongest relationship was between let-7b-5p and FP671120.3 (R = -0.342, adj p = 3.5E-15). The miRNA that displayed the largest number of relationships with other ncRNAs was miR-30a-5p. MBM ncRNAs generally clustered into two groups with extremely strong ([R] > 0.90) direct or indirect relationships. These clusters were generally comprised of ncRNAs that formed a common subunit (i.e., RNA5-8SN1, RNA5-8SN2, RNA5-8SN3).

## 4 Discussion

This study characterizes the most common miRNAs and ncRNAs across the four months after delivery. The results define relationships between miRNAs and other ncRNAs, and identify maternal characteristics that impact MBM miRNA content. A wide variety of miRNAs and ncRNAs are present in MBM. No single class of ncRNA predominates, with rRNAs, lncRNAs, miRNAs, and a mitochondrial RNA all among the top 17 ncRNA features. However, a small number of miRNAs account for the vast majority of miRNA content. These 11 “abundant” miRNAs overlap substantially with the most common miRNAs identified in previous studies (37). Their mRNA targets are involved in functions such as hormonal signaling and inflammation – supporting previous studies that have posited a role for MBM miRNAs in breast function, as well as immunoregulation (46, 50). In fact, the miRNA most abundant in MBM (miR-146b-5p, 28% of miRNA content) has been implicated in breast tissue proliferation, metabolic regulation, and the immunologic responses to upper airway infection (51–53).

The results demonstrate that milk maturity impacts MBM levels of ncRNAs. Levels of miRNAs generally increase as milk maturity increases, while other ncRNAs tend to decrease. MBM levels of ncRNAs are most similar at 1 and 4 months post-delivery. This finding is consistent with other studies that have detected minimal changes in MBM miRNA content of mature milk (16, 41, 42). However, in the current study, levels of ncRNAs differ substantially in the first week after delivery, compared with one month later. This contradicts previous studies that have found minimal evolution of miRNA content in the first month post-delivery (22, 42). The conflicting findings may be a result of the milk fraction being studied, the analytic technique used to measure miRNAs, or the additional power provided by this study’s large sample size.

Our study design provided a unique opportunity to determine if MBM RNA profiles were impacted by maternal characteristics. The results show that maternal characteristics do not have a large effect on the majority of miRNAs or other ncRNAs. Maternal diet impacts the largest number of ncRNAs. Among the 11 most abundant MBM miRNAs, 4 display an effect of dairy intake, 1 is affected by calcium intake, and 1 is affected by gestational diabetes. These results suggest modifications in maternal diet may be capable of shifting the MBM miRNA profile by altering abundant miRNAs.

Several other sparse miRNAs display significant effects of maternal age, BMI, parity, prior breastfeeding experience, and tobacco use. However, the majority of miRNAs are not impacted by these factors, and those that are impacted, generally display a low magnitude of effect. Among the most dynamic miRNAs is miR-196a-5p, which is lower among young primiparous mothers, and mothers with a history of tobacco use. Previous studies have demonstrated the importance of miR-196 in cell growth and differentiation (54, 55), highlighting a potential role for this miRNA in breast cancer (56, 57).

The strengths of the present study include: 1) a large sample size with longitudinal collections; 2) consistent procedures for sample collection/processing; 3) high throughput sequencing to simultaneously measure all known miRNAs and other ncRNA classes; and 4) the use of mixed effects models to assess relative impacts of maternal characteristics. There are several limitations. The current cohort was mostly white, and included only mothers delivering at term. This may limit generalizability of the findings. Our analysis focused on the milk lipid fraction. Prior studies have demonstrated minimal miRNA differences across MBM fractions (14, 20), however results in skim or cellular fractions may differ. Our results (which include both exosomal and non-exosomal RNA) may differ from studies focused solely on *exosomal* RNA (40). Although exosomal RNA has a higher likelihood of gastrointestinal absorption (58), prior studies have demonstrated absorption of non-exosomal miRNAs (59). Further, our analysis focuses on highly abundant RNAs which also increases likelihood of absorption.

Our analysis of collection time clustered samples into morning, afternoon, and nighttime groups. However, a cosine regression approach might detect subtle relationships between MBM miRNAs and time. MBM contains melatonin (60), which has immunomodulatory effects on infant sleep and behavior (61). Since miRNAs have diurnal variation and regulate circadian rhythm (62), it will be important for future studies to explore the potential relationship between MBM miRNAs and melatonin.

The results of this study advance our collective understanding of MBM RNA profiles by identifying miRNAs and other ncRNAs features most abundant in the four months after delivery. Although a few ncRNAs dominate the MBM profile, these miRNAs vary as a function of milk maturity and diet. Putative miRNA functions indicate they may play an important role in maternal breast function, or contribute to infant immune development and metabolism. Therefore, future studies should interrogate the relationship between MBM miRNAs and infant health outcomes such as obesity and atopy.

## Data Availability Statement

The data presented in the study are deposited in the Gene Expression Omnibus repository, accession number GSE192543. GEO respository link: https://www.ncbi.nlm.nih.gov/geo/query/acc.cgi?acc=GSE192543.

## Ethics Statement

The studies involving human participants were reviewed and approved by The Independent Review Board at the Penn State College of Medicine (STUDY00008657). Written informed consent to participate in this study was provided by the participants’ legal guardian/next of kin.

## Author Contributions

SH conceived of the study, designed the study, oversaw data collection and sample processing, completed the statistical analysis, and drafted the manuscript. AC aided study design and was responsible for data collection. KW was responsible for sample processing. DC aided in sample processing and data analysis. All authors provided critical revisions and approved of the submitted manuscript.

## Funding

This work was supported by grants from The Gerber Foundation [#204135], and The Center for Research on Women and Newborns (CROWN) Foundation [#48Z3] to SH. The funding sources had no role in the study design, the collection, analysis or interpretation of data, the writing of the manuscript; or the decision to submit the article for publication.

## Conflict of Interest

The authors declare that the research was conducted in the absence of any commercial or financial relationships that could be construed as a potential conflict of interest.

## Publisher’s Note

All claims expressed in this article are solely those of the authors and do not necessarily represent those of their affiliated organizations, or those of the publisher, the editors and the reviewers. Any product that may be evaluated in this article, or claim that may be made by its manufacturer, is not guaranteed or endorsed by the publisher.
