# Supplementary material for: Levels of Breast Milk MicroRNAs and Other Non-Coding RNAs Are Impacted by Milk Maturity and Maternal Diet
Source: Front Immunol. 2022 Jan 14;12:785217. doi: 10.3389/fimmu.2021.785217 (PMC8796169; doi:10.3389/fimmu.2021.785217)

Supplementary Material

**Figure S1. Sphericity analysis and sample normalization**

A principal components analysis was used to visualize two-dimensional projection of the 503 milk samples based on total RNA profiles (left image). The PCA accounted for 27.5 of the total variance in RNA data. There were three milk samples that clearly fell outside the 95% confidence interval, and these were removed from downstream analysis. Raw RNA counts were median normalized (right image). Distribution of counts across all RNA features is shown before and after normalization.


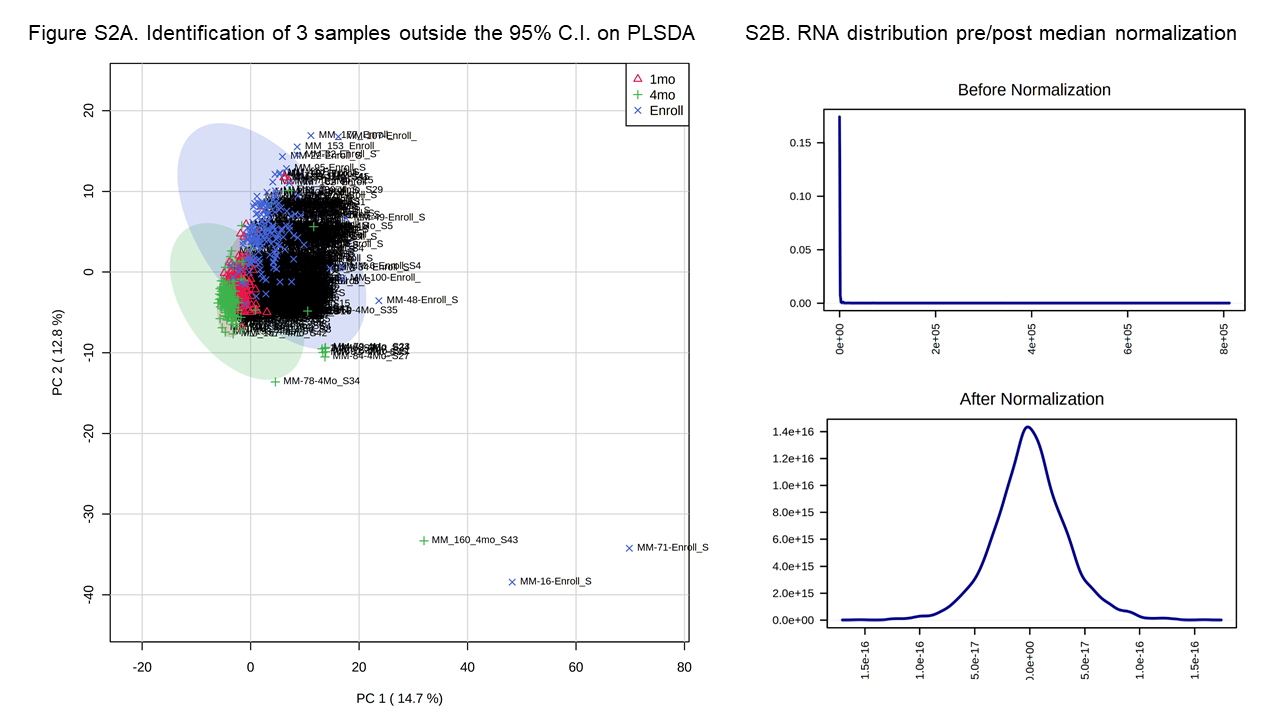


**Figure S2. Seven ncRNAs most significantly associated with maternal diet**

The scatterplots display 6 ncRNAs and 1 miRNA that demonstrated significant relationships (adj p < 0.05) with maternal diet (as measured by the Diet Survey Questionnaire). Maternal dairy intake at 0 months post-delivery was directly associated with MBM levels of DIS3 (R = 0.31, adj p = 0.34), DMXL1 (R = 0.30, adj p = 0.034), and TRPM2 (R = 0.30, adj p = 0.036). Maternal fruit intake at 0 months post-delivery was directly associated with MBM levels of CRIP2 (R = 0.32, adj p = 0.023), while fruit intake at 1 month post-delivery was associated with levels of HNRNPA1 (R = 0.33, adj p = 0.005). Maternal intake of sugar at 0 months post-delivery was directly associated with MBM levels of EIF3F (R = 0.38, adj p = 0.00022). Maternal intake of calcium at 1 month post-delivery was directly associated with MBM levels of miR-374b-3p (R = 0.27, adj p = 0.020).


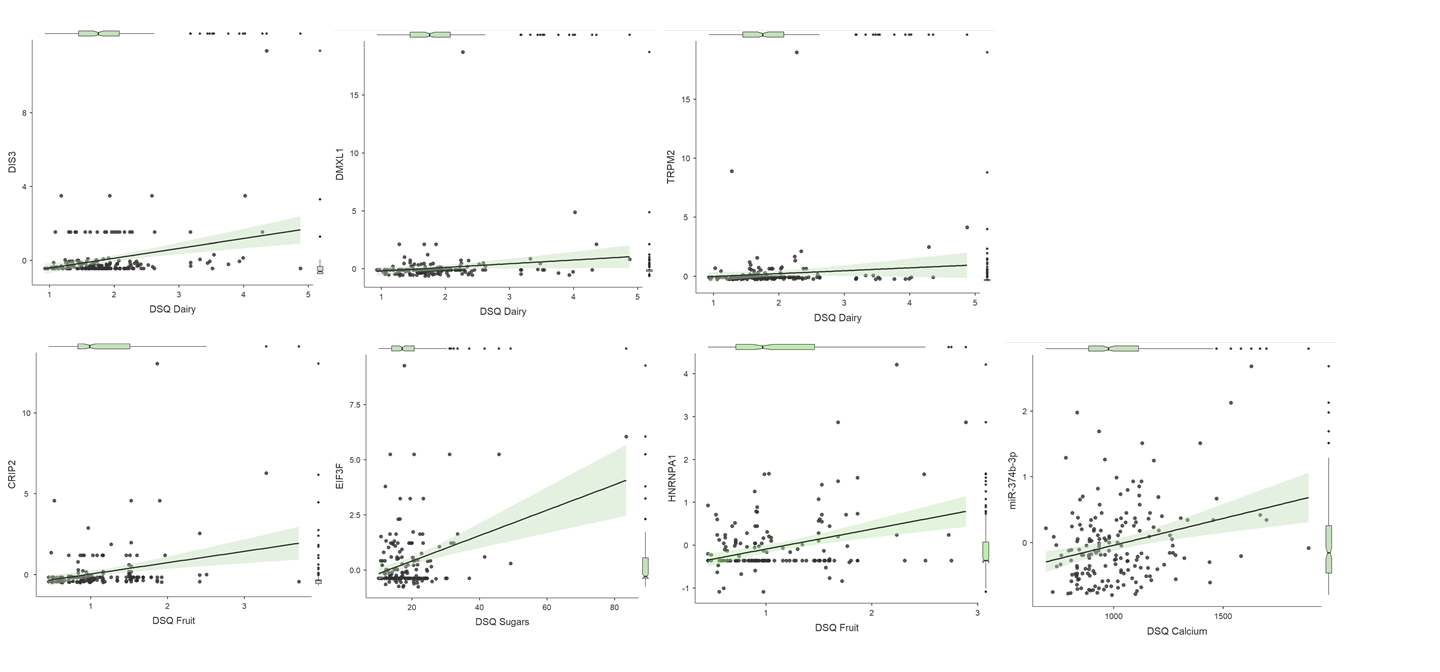


**Figure S3. MBM levels of six miRNAs one month post-delivery display relationships with previous breastfeeding experience**

The hierarchical clustering heatmap shows six miRNAs whose levels in MBM one month after delivery displayed a significant effect (adj p < 0.05) of previous breastfeeding experience (never breastfed (0), red; 0-1 months, green; 1-2 months, blue; 2-4 months, light blue; or 4+ months, pink). There were three miRNAs (miR-885-5p, miR-196a-5p, and miR-516a-5p) that were highest (dark red) in women who had previously breastfed for ≥ 4 months (pink group). The dendrogram uses a Pearson distance metric to demonstrate relatedness of the six miRNAs.


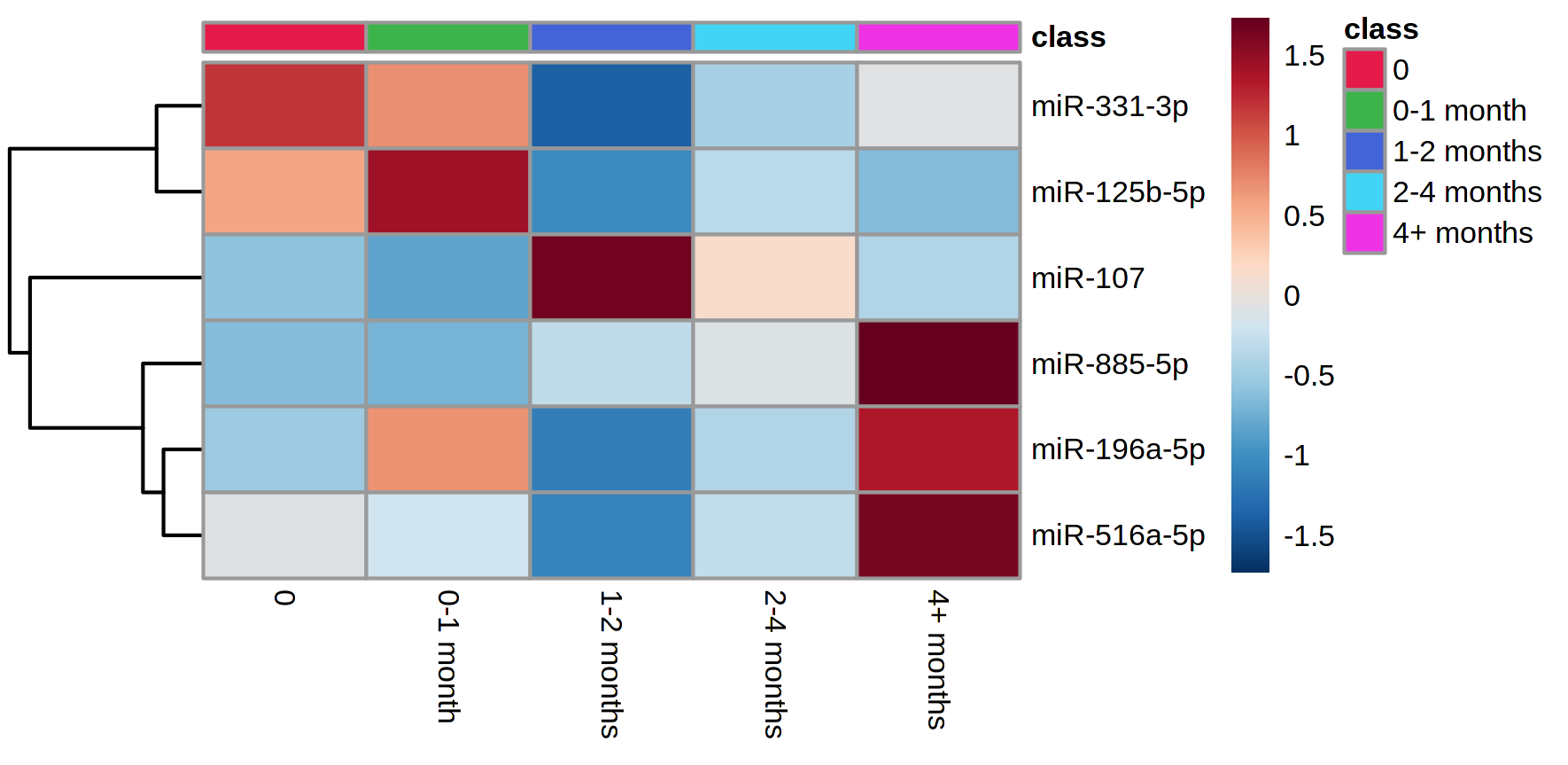


**Figure S4. Levels of miR-196a-5p are impacted by several maternal characteristics**

The levels of miR-196a-5p within maternal breast milk were affected by parity (*x*^2^ = 33.4, adj p = 7.2x10^-4^), maternal tobacco use (fold change = 0.118, adj p = 0.013), and maternal age (R = 0.28, adj p = 0.010). Levels of miR-196a-5p were lower in primiparous mothers (red) relative to mothers who had delivered previously (left figure). They were also lower in women with former tobacco use (red) relative to women who had never used tobacco (middle figure). Finally, levels of miR-196a-5p generally increased with maternal age (right figure).


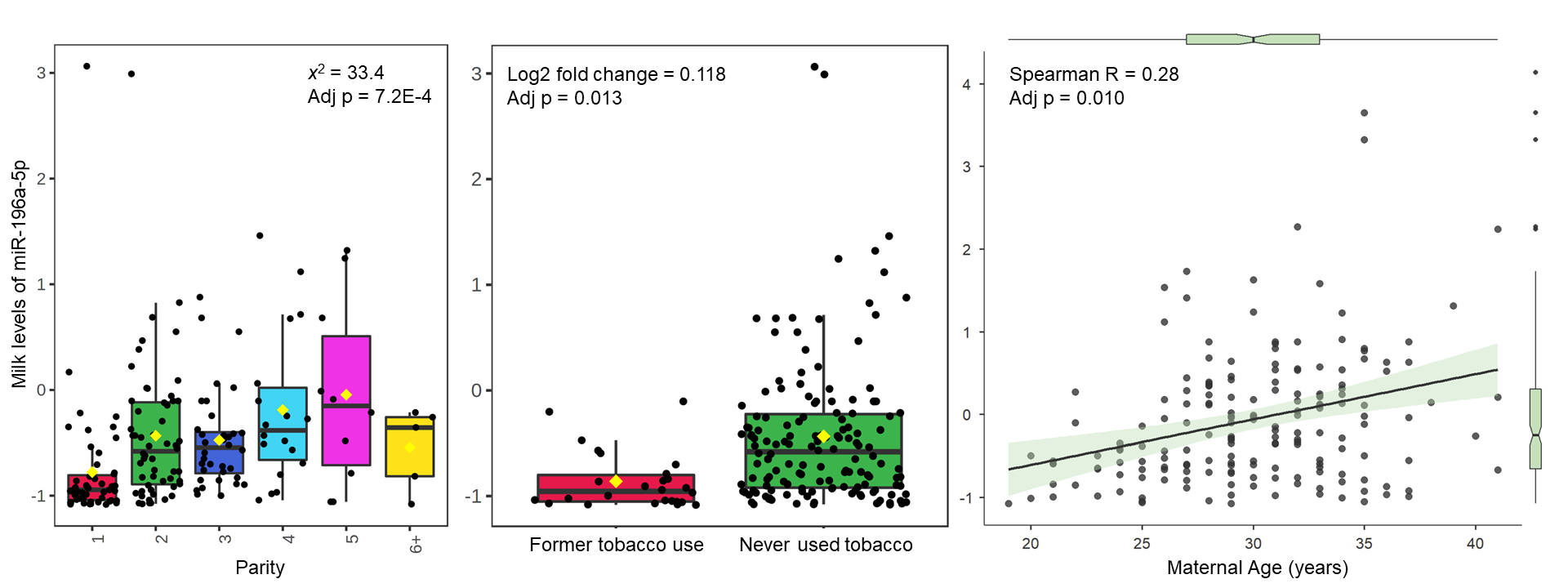

Supplement: Supplementary file 2 [file DataSheet_2.docx]
